# Malignant fibrous histiocytoma originating from the mesorectum: a case report

**DOI:** 10.1186/1477-7819-9-15

**Published:** 2011-02-02

**Authors:** Yoshifumi Nakayama, Noritaka Minagawa, Takayuki Torigoe, Koji Yamaguchi

**Affiliations:** 1 Department of Surgery 1, School of Medicine, University of Occupational and Environmental Health, Japan

## Abstract

**Background:**

Malignant fibrous histiocytoma (MFH) is a common sarcoma affecting soft tissues of the body, especially of the extremities or trunk. Prognosis of the abdominal MFH is usually poor.

**Case presentation:**

A 52-year-old female presented to our surgical outpatient clinic with a lower abdominal tumor that had been gradually increasing in size. Clinical examination revealed a firm, irregularly surfaced, fixed, painless, child-head-sized tumor located in her lower abdomen. Computed tomography (CT) and magnetic resonance imaging (MRI) of the abdomen revealed a polycystic tumor at the lower abdomen which was 15 × 13 × 11 cm in diameter and encased the colorectum to the left back side. A barium enema and a colonoscopy showed direct invasion to the rectum. In 2001, the tumor had been excised along with a low anterior resection of the rectum because of direct invasion. The origin of this tumor was the mesorectum. The weight of the excised tumor was 1,500 g, including 800 ml of a brown fluid. A histopathological diagnosis revealed a common type of MFH, in which mitotic figures are frequently seen.

**Conclusion:**

This patient has survived without recurrence, for approximately 8 years since the completed tumor resection. It is important to obtain a complete resection during the MFH treatment.

## Background

Malignant fibrous histiocytoma (MFH) is a common sarcoma affecting soft tissues of the body, especially of the extremities or trunk [1-3]. The tumor cells are derived from histiocytes capable of fibroblastic transformation [4]. MFH is an aggressive tumor with a high potential of demonstrating metastasis to other parts of the body. The prognosis of patients with abdominal MFH is usually poor [5].

Primary mesenteric MFH is a rare disease and few cases have been reported in the English literature [6-10]. We herein report a surgical case of MFH that originated from the mesorectum and affected the rectum.

## Case Report

A 52-year-old female presented to our surgical outpatient clinic with a lower abdominal tumor that had been gradually increasing in size. She first noticed a fist sized, painless tumor about four months ago. Clinical examination revealed a firm, irregularly surfaced, fixed, painless, child-head-sized tumor in her lower abdomen. Laboratory data showed that she had a white blood cell count of 5500/mm^3^, hemoglobin of 8.3 g/dl, hematocrit of 25.4%, platelets count of 429,000/mm^3^, normal electrolytes, as well as normal blood urea nitrogen levels and the liver function.

**Figure 1:**
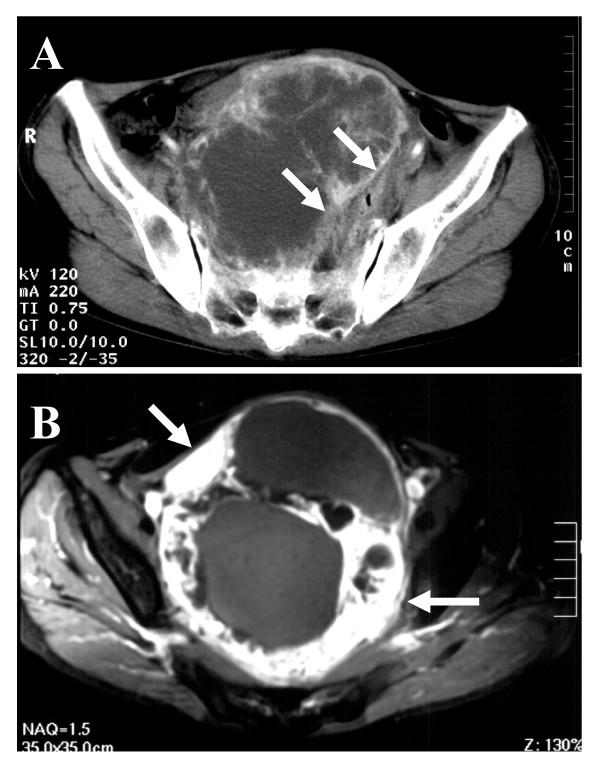
**A) An abdominal CT revealed a large uneven tumor in the lower abdomen and encased the colorectum to the left back side (*arrows*)**. B) An MRI of the lower abdomen revealed a polycystic tumor with a part of the thickened wall (*arrows*).

**Figure 2:**
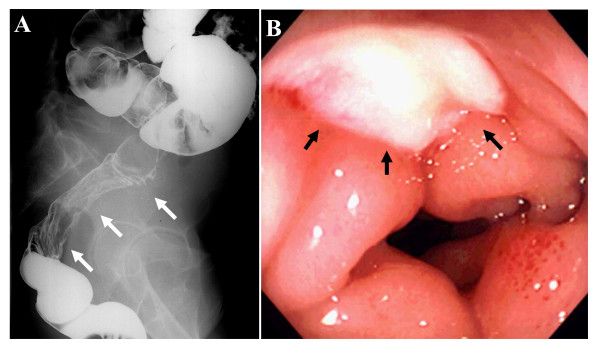
**A) A barium enema revealed an encasement of the sigmoid colon and rectum toward the left back side**. Some parts of the upper rectum had irregular mucosa (*arrows*). B) A colonoscopy revealed a round ulcer with a white coating (*arrows*).

Computed tomography (CT) of the abdomen demonstrated a large tumor in the lower abdomen which was 15 × 13 × 11 cm in diameter and encased the colorectum to the left back side (Figure 1A). Magnetic resonance imaging (MRI) of the lower abdomen indicated a polycystic tumor (Figure 1B). A barium enema revealed that the tumor had encased the rectum toward left posterior, and some parts of the upper rectum had irregular mucosa (Figure 2A). A colonoscopy revealed an encasement of the upper rectum and also round ulcers with a white coating, whose histological examination determined granulation tissue (Figure 2B).

**Figure 3:**
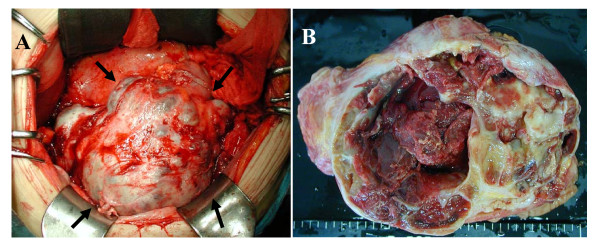
**A) A laparotomy revealed a child-head-sized tumor that encased the other organs (*arrows*)**. B) An operative specimen revealed a polycystic tumor with a part of the thickened wall.

**Figure 4:**
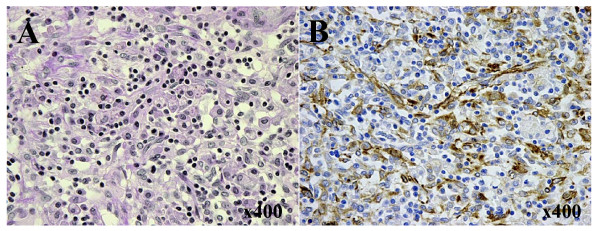
**A) A histopathological examination revealed pleomorphic cells proliferating in a storiform pattern (H & E, ×400)**. B) Tumor cells showing positive staining for vimentin (×400).

In 2001, this tumor was excised along with a low anterior resection of the rectum for the complete resection (Figure 3A, B). The mesorectum including the tumor was well circumscribed from surrounding organs, with the exception of the rectum. Therefore, the origin of this tumor was thought to be the mesorectum. The uterus and the ovaries had no local lesions. The weight of the excised tumor was 1,500 g. This tumor included 800 ml of a brown fluid, whose cytological examination determined class I. A histopathological examination revealed proliferation of pleomorphic cells in a storiform pattern (Figure 4A). Mitotic figures were also frequently observed. Immunohistochemical analyses indicated that many of the tumor cells were positive for vimentin (Figure 4B), while tumor cells were negative for cytokeratins, desmin, S-100 protein, actins, c-kit, and CD34. These features are compatible with MFH of a common type.

The postoperative course was uneventful, and she left the hospital on the 15th postoperative day. A postoperative follow-up CT revealed no recurrence of the MFH in any organs since the resection was completed.

## Discussion

Retroperioneal and mesenteric MFH have been reported to occur in the rate from 5.7% to 16% of the total MFHs cases [1,2,5,11]. Several reports have so far indicated that MFH derived from the retroperitoneum includes mesenteric MFH because it is difficult to determine the true origin of the huge tumor. Due to this condition, mesenteric MFH is very rarely observed [6-10]. In our case, the tumor originated from the mesorectum because the huge tumor was limited to within the capsule of the mesorectum.

MFH is an aggressive tumor with a high potential of metastasis to other parts of the body. The rates of local recurrence and distant metastasis are 44% and 42%, respectively [1]. The most frequent metastatic site is the lung (82%), followed by the lymph nodes (32%) [1]. Although the 5-year survival rate of all MFH patients that underwent surgery is 67.2% [11], the 5-year survival rate of patients with abdominal MFH is reportedly 14% [5]. On the other hand, the 5-year survival rate of patients with mesenteric MFH has not been reported because the disease is extremely rare. The current treatment of choice for primary MFH is surgical resection which means wide excision of the tumor with an aim for a tumor-free margin. Presently, the efficacy of adjuvant chemotherapy or radiation has not been clear in the case of retroperitoneal and visceral MFH. Therefore, this patient received neither adjuvant chemotherapy nor radiation.

Mesenteric MFH initially grows without any significant clinical symptoms, and finally produces clinical symptoms and signs later when it compresses or invades the adjacent organs [9]. In this case, she noticed the lower abdominal tumor without any symptoms: nevertheless, the tumor size was 15 × 13 × 11 cm and the tumor had directly invaded to the rectum. Therefore, a complete tumor resection combined with a low anterior resection of the rectum was needed. A previous report indicated that colonic lesions of retroperitoneal MFH were detected by a barium enema and colonoscopic examination before the laparotomy [12]. In the present case, rectal ulcers were also identified by a barium enema and colonoscopic examination before the laparotomy. Moreover, a histologic examination of the resected specimen clearly demonstrated a submucosal invasion by the MFH. It is very important that the requirement of the combined resection with other organs is decided before the operation begins.

The efficacy of chemotherapy against advanced or metastatic MFH is still unclear. On the other hand, several phase studies which investigate the efficacy of chemotherapy against advanced or metastatic soft tissue sarcoma including MFH, have been reported [13-16]. These studies indicated that doxorubicin and ifosfamide are the most active agents for the treatment of this clinical condition [13-16]. However, the response rate of these agents has been reported to range between 10% and 36%, and the median overall survival of these agents has been described to be between 10 and 12 months [13-16]. Therefore, in order to obtain an improvement in the survival in the patients with soft tissue sarcoma including MFH, further investigation of the new available agents is thus called for.

## Conclusion

This patient has survived without recurrence for approximately 8 years after the completed tumor resection. A complete resection is important to obtain a favorable prognosis for MFH.

## Consent

Written informed consent was obtained from the patient for publication of this case report and accompanying images. A copy of the written consent is available for review by the Editor-in-Chief of this journal.

## Competing interests

The authors declare that they have no competing interests.

## Authors' contributions

NY, MN, TT and YK contributed equally to this work; NY designed the research; NY, MN and TT performed and analyzed the data; NY and YK wrote the paper.
